# Pharmacist-led migraine consultations in community pharmacies: a pilot study of pharmacists’ experiences and patient-reported outcomes

**DOI:** 10.1186/s10194-026-02374-3

**Published:** 2026-04-29

**Authors:** Lene Berge Holm, Sahar Hussain, Parisa Gazerani, Tonje Krogstad

**Affiliations:** 1 Department of Life Science and Health, Faculty of Health Sciences, Oslo Metropolitan University, Oslo, Norway; 2 Health Services Research Unit, Akershus University Hospital, Lørenskog, Norway

**Keywords:** Pharmacist-led consultations, Migraine, Patient-reported consultation experience, Patient education, Community pharmacy, Pilot study

## Abstract

**Background:**

Migraine is a prevalent neurological disorder and a leading cause of disability worldwide. Despite effective medications, many patients experience underdiagnosis, suboptimal medication use, and poor adherence. Pharmacists are well-positioned to provide medication counselling, yet their role in structured migraine counselling services within community pharmacy practice remains underexplored.

**Objective:**

This pilot study explored pharmacists’ experiences and patient-reported consultation outcomes associated with a pharmacist-led migraine consultation service in community pharmacies, with the aim of generating exploratory evidence to inform future implementation.

**Methods:**

A mixed-methods prospective pilot service evaluation was conducted, and findings were interpreted using an implementation science perspective. Pharmacists from 19 Norwegian community pharmacies received targeted training and conducted structured migraine consultations between May 2023 and January 2024. Each patient was offered two consultations. In total, 198 first consultations and 44 follow-up consultations were completed. Semi-structured pharmacist interviews were analysed qualitatively, and patient-reported motivation for and experiences with the consultations, use of recommendations, and changes in frequency and/or severity of migraine attacks were assessed using self-reported and pharmacist-reported questionnaires. Chi-square tests and Wilcoxon signed-rank tests were used to explore differences between observations.

**Results:**

Pharmacists reported that migraine consultations were perceived by pharmacists as implementable within pharmacy practice, professionally valuable, and strengthened their role in primary healthcare. Time constraints and funding were noted as barriers. Overall patients reported positive consultation experiences (adjusted *p* < 0.011). Among patients returning for follow-up consultations, 69% reported having implemented at least one recommendation from the pharmacist at the first consultation, and 38% reported perceived improvement in migraine status. Participants who reported implementing recommendations tended to report fewer and/or less severe migraine attacks; however, this difference did not reach statistical significance (*p* = 0.085). Motivation for medication use was high both before and after the consultations.

**Conclusion:**

This pilot service evaluation suggests that pharmacist-led migraine counselling may represent a clinically relevant and potentially useful addition to community pharmacy practice. Responding patients reported positive consultation experiences and reported to be highly motivated to take their medication. The majority implemented at least one recommendation, and many reported perceived improvements in migraine status. However, limited follow-up data indicates that findings should be interpreted cautiously. Larger-scale studies with validated instruments are needed to assess long-term effects and implementation models.

## Graphical Abstract

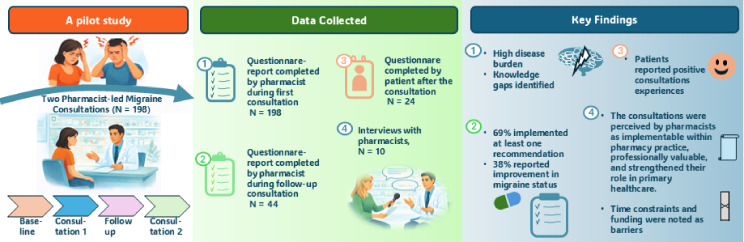

## Introduction

Migraine is a highly prevalent neurological disorder affecting over one billion people globally and remains one of the leading causes of years lived with disability (YLDs), particularly among individuals of working age [1]. Despite well-established treatment guidelines and the availability of acute and preventive medications, migraine management remains suboptimal in both primary and specialist healthcare [2]. Many patients experience underdiagnosis, delayed initiation of treatment, incorrect medication use, or poor adherence to prescribed therapies [3]. A substantial number rely on self-medication with over-the-counter analgesics or prescription drugs such as triptans without receiving sufficient guidance on correct use [4]. This may lead to medication-overuse headache (MOH), characterised by increasing headache frequency and reduced treatment efficacy [5].

Adherence to preventive migraine medication is especially low. Studies show that fewer than half of patients continue prophylactic treatment beyond six months [6]. Contributors to poor adherence include limited patient knowledge, unrealistic expectations of therapeutic outcomes, concerns about side effects, lack of follow-up, and insufficient guidance from healthcare providers [7]. Furthermore, optimal migraine management extends beyond pharmacotherapy: it involves recognising triggers, timing medication intake relative to symptom onset, maintaining sleep and lifestyle routines, and understanding when to escalate treatment or seek specialist evaluation [8]. Without structured counselling, patients frequently resort to trial-and-error strategies, resulting in ineffective treatment patterns, unnecessary suffering, and reduced quality of life [9]. These challenges highlight the need for structured, accessible counselling approaches that support appropriate medication use in routine care settings.

Community pharmacists are among the most accessible healthcare professionals and regularly interact with migraine patients seeking both prescription and over-the-counter medication [10]. Unlike general practitioners, pharmacists are available without appointment and can provide rapid access to medication counselling, follow-up, and monitoring. Pharmacist-led interventions in chronic diseases such as asthma, hypertension, diabetes, and chronic pain have demonstrated improved medication adherence, self-management, and clinical outcomes [11]. However, the role of pharmacists in migraine-specific counselling has received limited empirical attention, and structured frameworks for implementing migraine counselling in pharmacy practice are lacking [12]. Understanding how such services function in real-world pharmacy settings is an important first step toward future implementation.

Existing studies suggest that while pharmacists feel confident providing general medication advice, many report insufficient training in neurological disorders such as migraine and uncertainty about offering disease-specific counselling [13]. There is also limited knowledge about how pharmacist-led migraine consultations are experienced by pharmacists and patients and how they may influence patient behaviour in routine practice [14]. Additionally, no studies have explored how structured migraine consultations can be integrated into everyday pharmacy workflow, nor how data from repeated consultations can be linked and evaluated in real-world practice [12].

Understanding how new healthcare services function in real-world practice requires consideration of both implementation-related factors and service or clinical outcomes. Implementation-related factors include aspects such as organisational context, professional roles, and workflow integration whereas service outcomes may include patient-reported experiences, behavioural changes, or perceived symptom improvement [15]. Implementation science frameworks, such as the Consolidated Framework for Implementation Research (CFIR) [16], provide structured approaches for identifying contextual determinants that may influence the introduction and delivery of new services in healthcare settings.

In this study, we conducted a prospective pilot service evaluation exploring pharmacists’ experiences and patient-reported consultation outcomes associated with a pharmacist-led migraine consultation service in Norwegian community pharmacies. The service consisted of two structured consultations focusing on medication use, dosing and timing, treatment expectations, trigger management, and adherence strategies. Pharmacists received targeted training before implementation. Each participating patient was offered two consultations.

The objectives of this pilot study were to:

1. Investigate pharmacists’ experiences with delivering migraine consultations, including perceived benefits, challenges, and training needs.
2. Identify medication-related informational needs and knowledge gaps among migraine patients regarding medication use and self-management.
3. Explore patients’ motivation for and experiences with pharmacist-led migraine consultations, implementation of recommendations, and patient-reported perceived changes in frequency and/or severity of migraine attacks and absence from work, school, and social activities after the consultations.

As a pilot study, the primary focus was to generate exploratory insights into implementation-related experiences and early service outcomes rather than to test effectiveness. We hypothesized that pharmacist-led migraine consultations would (H1) strengthen pharmacists’ perceived confidence and professional relevance, (H2) be associated with increased patient- reported understanding of medication use (including dosing, timing, and expectations), (H3) be associated with positive patient-reported consultation experiences, and (H4) be associated with self-reported changes in migraine burden (e.g., severity and frequency) and participation or absence from work, school, and social activities.

By generating early evidence from real-world pharmacy practice, this pilot study lays the groundwork for larger, controlled studies and contributes to the development of structured migraine consultation models within community pharmacies. Findings from this pilot evaluation may inform future development and implementation of structured migraine consultation models within community pharmacies. Such interventions may support improved migraine self-management, reduce medication overuse, and strengthen pharmacists’ role in collaborative primary healthcare.

## Methods

### Study design

This study employed a mixed-methods prospective pilot service evaluation design to explore implementation-related experiences and preliminary service outcomes of pharmacist-led migraine consultations in Norwegian community pharmacies. The counselling service and consultation guide was developed within pharmacy practice in collaboration with Apotek 1 and clinical stakeholders prior to the present study, while the academic research team conducted an independent scientific evaluation of its implementation and outcomes. The quantitative component comprised two data sources. Patient-reported data were collected after the consultations through a questionnaire with closed-ended questions assessing motivation for and experiences with the consultations, as well as motivation for medication use. Pharmacist-reported data were collected through questionnaires completed during the migraine consultations and included identified medication related knowledge gaps, implementation of recommendations from the initial consultation, self-reported changes in migraine attack frequency and/or severity, and changes in absence from work, school, or other daily activities. Data were collected across two structured consultations. The qualitative component of the study was limited to semi-structured interviews with pharmacists and explored pharmacists’ experiences, perceived challenges, and the influence of the consultations on their professional practice.

The quantitative component followed a longitudinal follow up within the service evaluation design, while the qualitative component was informed by a phenomenological approach.

Although the study was not prospectively designed using an implementation framework, findings were retrospectively interpreted using CFIR [16] to support structured understanding of implementation-related determinants and experiences.

The study was reported in accordance with the STROBE guidelines [17] for observational research and the COREQ checklist [18] for qualitative studies. As a pilot study, the primary focus was to generate exploratory insights into implementation-related experiences and early service outcomes rather than to test effectiveness.

### Setting and participants

The study was conducted in community pharmacies belonging to the Apotek 1 pharmacy chain in Norway, where the migraine counselling service is being tested for potential future implementation as part of routine pharmacy practice. Pharmacists were recruited from 19 pharmacies located in urban, suburban, and rural regions of Norway. To be eligible, pharmacists were required to hold a valid license, to have completed a structured training programme in migraine pharmacotherapy and patient counselling, and to have participated in delivering at least two migraine consultations before or during the study period. Recruitment was conducted through regional pharmacy managers, professional networks, and direct invitations, with purposive sampling to ensure variation in years of professional experience, geographic location, and pharmacy size.

Patients were invited to participate when they approached the pharmacy for migraine-related medication or advice. Inclusion criteria were being 18 years or older, having a self-reported or physician-confirmed diagnosis of migraine, and having current or previous experience with migraine medications, including acute and preventive treatments such as triptans, non-steroidal anti-inflammatory drugs, gepants, or preventive therapies. Patients were excluded if they had significant cognitive impairment, were unable to understand Norwegian, or had comorbidities that could severely interfere with medication adherence. Those who agreed to participate were offered two consultations and provided written informed consent.

### Pharmacist-led migraine consultations service

The pilot service consisted of structured counselling delivered within routine community pharmacy practice. It was explored as a potential model for future integration into regular pharmacy services and comprised two structured individual consultations delivered in designated consultation rooms within pharmacies, or by phone. To ensure consistency across sites, a standardized consultation guide, developed collaboratively by pharmacists, researchers, and migraine specialists, was used. Pharmacists completed a structured training program developed by Apotek 1 prior to implementation of the counselling service. The training lasted approximately five hours in total and consisted of digital self-learning modules combined with instructor-led sessions. The program covered migraine pharmacotherapy, correct timing and dosing of triptans and other migraine medicines, prevention of medication-overuse headache, use of the standardized counselling guide, documentation procedures, and practical communication scenarios. Pharmacists were required to complete all training components prior to delivering the counselling consultations.

During the first consultation pharmacists collected demographic characteristics (age, sex, and education level), and information about migraine history, including attack frequency, medication use, and potential triggers. They also assessed patients’ medication related knowledge using predefined questionnaire items addressing medication administration, dosing, side effects, interactions, and expected effects. They reviewed acute and preventive treatments and provided education on correct timing, dosing, expected treatment effects, and potential side effects of medications. Particular emphasis was placed on the appropriate use of triptans and the prevention of medication-overuse headache. Patients were also introduced to the use of headache diaries and advised on lifestyle measures and when to consult a physician.

The second consultation, which typically occurred between two and eight weeks after the first one, focused on evaluating changes from the initial consultation. Pharmacists discussed whether patients had followed recommendations, whether they had experienced changes in migraine attack frequency and/or severity after the first consultation, and whether they had experienced a change in absence from work, school, or social activities. No automated reminder system was used; therefore, attendance at the follow up consultation depended on patient initiative.

### Data collection

Qualitative data were collected through semi-structured interviews with pharmacists after they had delivered at least two consultations. Interviews were conducted via secure video conferencing, lasted between 20 and 40 min, and were audio recorded with consent before being transcribed verbatim. The interviews started with a brief introduction, including information about the interviewer and her interests and roles in the research project, and the aims of the study. Interview topics included experiences with counselling implementation, perceived patient needs, professional development, and practical barriers such as time pressure and workflow constraints. Two pilot interviews were conducted, but only minor changes were made to the interview guide after these. Thus, the results from these interviews were included. No open-ended questionnaire responses were included in qualitative analyses, as all questionnaire items used in this evaluation consisted of structured closed-ended responses solely analysed quantitatively.

Quantitative data were obtained from patient encounters during both consultations. These data were collected with the tool Questback. Three different questionnaires were used for first consultations for new users, first consultations for experienced users, and for the follow-up consultations. Patients were categorized as first-time or inexperienced users if they reported no or limited prior experience using migraine medication, whereas experienced users were defined as patients who had previously used migraine medication prior to the consultation.

The assessments used in this pilot evaluation reflected patients’ responses recorded by the pharmacist during the consultations using structured questionnaire items rather than open-ended pharmacist evaluations.

Following the consultation, patients were invited to complete a questionnaire assessing motivation and consultation experiences using structured, closed-ended response options to be analysed quantitatively.

The questionnaires were originally developed as part of the operational migraine counselling service and were intended for service evaluation purposes rather than as psychometrically validated research instruments.

### Data analysis

Qualitative data were analysed using systematic text condensation as described by Malterud [19], a pragmatic, descriptive method inspired by phenomenological principles and well suited for exploratory health services research. The approach was applied to identify and summarise pharmacists’ experiences rather than to develop theory, in line with the exploratory nature of this pilot study. Two researchers independently reviewed and manually coded the transcripts to ensure credibility. Discrepancies were resolved through discussion, and analytical themes were refined iteratively.

Quantitative data were analysed using SPSS version 31.0.0.0(117), and Excel version 2507. Descriptive statistics were used to summarise participant characteristics, baseline migraine status, and patient medication related knowledge gaps. A Chi-square test was used to estimate the correlation between the use of advice after the first consultation, and improvement in migraine frequency and/or severity. Patient-reported consultation experiences were estimated through six items, including perceived learning about medication use and rationale, having questions answered, support in preventing side effects, reduced concerns following the consultation, and receiving advice on remembering to take medications. Responses were recorded on a five-point Likert scale ranging from strongly agree to strongly disagree. Each item was tested against the neutral midpoint of the scale using one-sample Wilcoxon signed-rank tests, where p-values were adjusted to account for multiple testing. A paired Wilcoxon signed-rank test was used to explore associations in motivation for medication use before and after consultations. Missing values were excluded from the analysis. Given the exploratory nature of the pilot study, and since the instruments were not validated, analyses were interpreted descriptively and cautiously.

## Results

### Pharmacists’ experiences with migraine counselling

Ten pharmacists from community pharmacies across Norway participated in the qualitative part of this pilot study. Two were men, and eight were women, aged between 26 and 48, with professional experience in pharmacy practice ranging from one to 20 years. Their demographic characteristics are presented in Table 1.

**Table 1:** Characteristics of pharmacists participating in the interviews

Interview number	Age	Gender	Educational background	Years of experience in Norwegian pharmacies	Duration of interview (min: sec)	Interview type
1	42	Female	MSc in Pharmacy	1	42:31	Pilot
2	31	Male	BSc in Pharmacy	1	24:29	Pilot
3	48	Female	BSc in Pharmacy	20	26:08	Main
4	48	Female	MSc in Pharmacy	20	31:12	Main
5	26	Female	MSc in Pharmacy	2	35:50	Main
6	28	Female	BSc in Pharmacy	4	32:36	Main
7	34	Female	MSc in Pharmacy	7	32:31	Main
8	26	Female	MSc in Pharmacy	8	27:59	Main
9	36	Female	MSc in Pharmacy	6	34:33	Main
10	46	Male	MSc in Pharmacy	12–13	34:58	Main

**Fig. 1:**
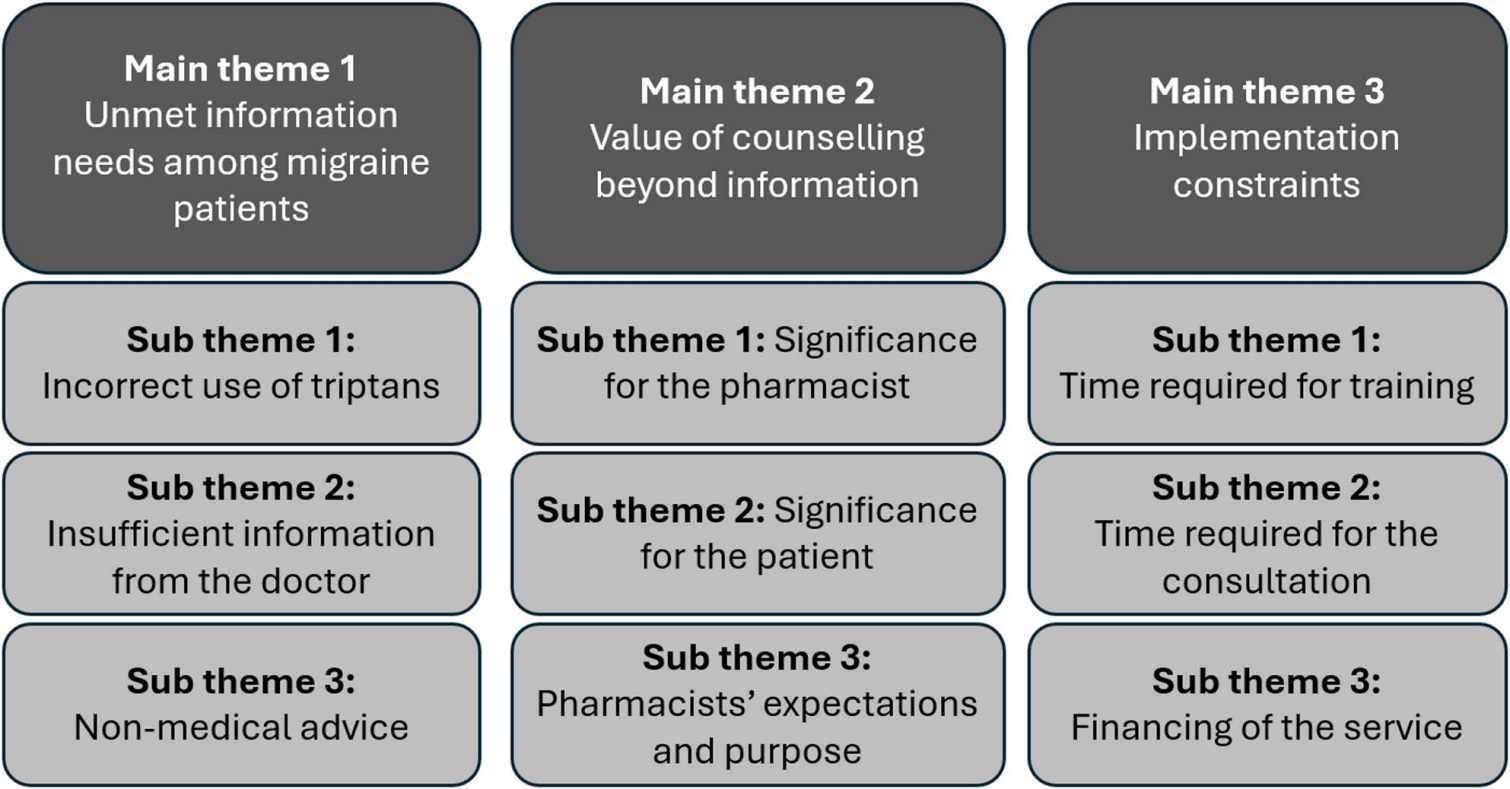
Analysis report after systematic text condensation

Analysis of the interview data resulted in three overarching themes: (1) Unmet information needs among migraine patients, (2) Value of counselling beyond information, and (3) Implementation constraints, each with three subthemes (Fig. 1). These themes reflect key implementation-related determinants, including perceived patient needs, professional role development, and organizational and workflow factors influencing service delivery.

#### Unmet information needs among patients

Pharmacists consistently reported that migraine patients lacked sufficient knowledge about medication use, particularly in relation to triptans. This applied to both newly diagnosed and experienced patients. Many patients were unsure about when to take triptans in relation to the onset of migraine symptoms, how frequently they could be used, whether different formulations could be alternated, and how to act if one medication was ineffective. One pharmacist explained:

*Most people may think that they must use a triptan over a fixed period of time. These patients are unsure whether they can switch to another triptan or whether they can combine these.”*

(Pharmacist 10)

Several pharmacists highlighted that patients often did not receive sufficient information or follow-up from their general practitioners. Prescriptions were frequently issued without consultation or counselling, leaving pharmacists to fill the knowledge gap. As one pharmacist stated:

*“I see that many migraine patients have not received good help from the doctor*,* and the patients may not know where they can get help. So they are simply prescribed triptans again and again*,* without having talked to the doctor directly…”.*

(Pharmacist 8)

Beyond medication use, pharmacists also provided patients with advice on non-pharmacological strategies such as lifestyle modifications, identifying individual triggers, and using headache diaries. These tools helped patients monitor attack frequency, triggers, and treatment responses, and were perceived to improve patient self-understanding and communication with healthcare providers. One pharmacist commented:

Most patients are positive about trying something new, especially when they realise that what they have been doing so far isn’t working. They want measures that can give them better disease control.

(Pharmacist 5)

#### Value of counselling beyond information

Pharmacists emphasized that migraine consultation was valuable not only for patients, but also for their own professional development and identity. The counselling service enabled them to utilise their pharmaceutical expertise more fully, reassure patients about correct medication use, and engage in meaningful patient follow-up. Patients reportedly appreciated being listened to and receiving personalized advice, which strengthened pharmacist–patient relationships. As one pharmacist reflected:

*«… I have a slight feeling that customers listen to specialists*,* the GP when they have a connection. But*,* now the patients also get to see what we can do…"*.

(Pharmacist 7)

These consultations also have value for the migraine patients beyond just receiving advice:

They feel that they are being listened to, and they appreciate that someone takes the time to hear what they have to say.

(Pharmacist 5)

Pharmacists also reflected on the purpose of their professional role, describing high expectations and motivation to contribute professionally through this programme. They described increased confidence in managing migraine-related consultations following participation in the programme and they reported feeling better equipped to provide structured counselling and to address patient questions regarding medication use and treatment expectations:

I expected that we would become professionally stronger in migraine, use our knowledge to help patients with the right advice, and make a real difference.

(Pharmacist 4)

#### Implementation constraints

Despite generally very positive attitudes towards pharmacist-led migraine consultations as a service, pharmacists highlighted barriers related to time use and resource allocation. Pharmacists described challenges related to completing training alongside routine duties, and ensuring patient return for follow-up consultations. These factors were perceived as practical organizational and workflow barriers to integrating the counselling service into everyday pharmacy workflow.

Pharmacists emphasised that the initial training required a substantial time investment from both pharmacies and individual staff members, and that motivation among employees was essential. One pharmacist described the training process as demanding and difficult to complete alongside regular work tasks:

It took a huge amount of time and made many pharmacists who started the training feel like they would never actually finish (…) If this is going to be a service, the training needs to be optimised. Too much time has to be set aside.

(Pharmacist 1)

The migraine consultation itself was also perceived as time-consuming compared with other pharmacy-based consultations. As one pharmacist explained:

I think time use is a challenge. It takes longer than other consultations we provide.

(Pharmacist 3)

To make the service realistically implementable in community pharmacies, pharmacists stressed the necessity of adequate financing that would allow both pharmacies and staff to allocate sufficient time. This was illustrated by the following quote:

On average, we have found that we spend much more time - around 40–45 min - on preparation, the consultation itself, and follow-up work. That has to be taken into account. Especially when it comes to reimbursement.

(Pharmacist 3)

### Migraine patients’ consultation experience and reported outcomes

Between May 2023 and January 2024, pharmacists across participating pharmacies conducted a total of 242 migraine consultations. Of these, 198 were first consultations, and 44 were follow-up consultations. A total of 24 patients completed the questionnaire about patient-reported consultation experiences and motivation for medication use after the consultation. This reflects a low voluntary response rate rather than a predefined sampling procedure, and analyses were therefore interpreted cautiously.

#### Patient characteristics, migraine burden, and medication related knowledge gaps

The majority of patients attending the first consultation were experienced users of migraine medications (*n* = 155), while 43 patients were first-time or inexperienced users. Demographic characteristics, including age, gender and education distribution for first consultation’s participants, are presented in Table 2. During the first consultation, patients were asked to report the number of migraine attacks and absence from work, school, or social activities experienced in the previous month. This, as well as patients’ medication related knowledge gaps, are also presented in Table 2. A high proportion of the patients, both experienced and new, reported more than eight attacks in the past 30 days, and more than half of the participants experienced absence from work, school or social activities, indicating a high frequency of migraine episodes and significant disease burden.

**Table 2:** Patient characteristics and migraine burden

	First time users, *N* = 43	Experienced users, *N* = 155
Age	18–25 years: 7 (16%) 26–44 years: 26 (60%) 45–59 years: 8 (19%) 60 years or older: 2 (5%) No valid response: 0	18–25 years: 18 (12%) 26–44 years: 60 (39%) 45–59 years: 54 (35%) 60 years or older: 22 (14%) No valid response: 1
Gender	Female: 36 (84%) Male: 7 (16%) Other or no valid response: 0	Female: 125 (81%) Male: 29 (19%) Other or no valid response: 1
Education	No education: 0 Elementary school: 0 High school: 19 (51%) College/university: 18 (49%) No valid response: 6	No education: 2 (1%) Elementary school: 4 (3%) Highschool: 55 (40%) College/university: 77 (56%) No valid response: 17
Number of migraine attacks experienced in the previous month	0–3 times: 14 (34%) 4–7 times: 10 (24%) 8 times or more: 17 (41%) No valid response: 2	0–3 times: 47 (31%) 4–7 times: 47 (31%) 8 times or more: 56 (37%) No valid response: 5
Absence from work, school, or social activities due to migraine during the past month	Yes: 28 (72%) No: 11 (28%) No valid response: 4	Yes: 84 (58%) No: 61 (42%) No valid response: 10
Self-reported medication knowledge	• Medication administration: Yes: 27 (90%) No: 3 (10%) No valid response: 13	• Medication administration: Yes: 136 (94%) No: 8 (8%) No valid response: 11
	• Dosing: Yes: 22 (73%) No: 8 (27%) No valid response: 13	• Dosing: Yes: 114 (79%) No: 30 (21%) No valid response: 11
	• Side effects Yes: 9 (30%) No: 21 (70%) No valid response: 13	• Side effects Yes: 68 (47%) No: 76 (53%) No valid response: 11
	• Interactions: Yes: 3 (10%) No: 27 (90%) No valid response: 13	• Interactions: Yes: 47 (33%) No: 97 (67%) No valid response: 11
	• Expected effects: Yes: 12 (40%) No: 18 (60%) No valid response: 13	• Expected effects: Yes: 82 (57%) No: 62 (43%) No valid response: 11

Data reported in the first consultation, number of responders and percentage of valid responses in parathesis.

The medication related knowledge gaps identified in the quantitative data were supported by findings from the qualitative interviews with pharmacists. During the interviews, pharmacists described that, based on their experiences from the consultations, patients exhibited substantial gaps in knowledge, including misunderstandings related to:

- The optimal timing of triptan administration relative to migraine onset (many patients delayed use until pain was severe).
- Correct dosage intervals and maximum daily or monthly triptan use, leading to concerns about misuse or risk of medication-overuse headache.
- Differences between acute and preventive treatments, and uncertainty about when preventive medication should be introduced.
- Medication expectations, with some patients expecting immediate pain relief and discontinuing treatment prematurely if the effect was delayed.
- Potential side effects and drug interactions, particularly fears of dependency or long-term harm.

Pharmacists reported that many patients also lacked knowledge of non-pharmacological strategies such as trigger identification, sleep regulation, hydration, stress reduction, and the use of headache diaries. Headache diaries were introduced as a tool to record symptoms, aura, stress, sleep patterns, medication intake, and triggers. Patients who used these diaries were perceived to develop a better awareness of their condition and communicate more effectively with healthcare providers.

#### Patient motivation and consultation experience

After the consultations, patients were encouraged to complete a self-report questionnaire about their motivation for and experiences with the consultation. Responses from 24 participants (4 men, 20 women) indicated that the most common motivations for participating in pharmacist-led consultations were the desire to learn more about migraine medication, and to improve medication technique. On a scale from 1 = very little motivation to 5 = highly motivated, the median response on motivation for using medications both before and after the consultations was 5. A Wilcoxon signed rank test for paired samples did not show any significant change in motivation score before and after the consultations (*p* = 0.167).

Patients’ perceived gains from the consultations were estimated through seven questions about perceived consultation experiences (question 1–7), three questions about the perceived quality and usefulness of the consultations (question 8–10), and one overall question (question 11). Table 3 presents the results from one-sample Wilcoxon signed rank tests where scores are compared to a neutral mid-point. Original and multiplicity-adjusted p-values (Bonferroni, k = 11) are presented for all 11 questions. These results indicate generally positive patient-reported consultation experiences and perceived quality of the pharmacist led migraine consultations.

**Table 3:** Patient motivation and experiences with consultations

Question	Response categories for the analyses. 1 = highly disagree 5 = highly agree Number of responders (percentage of valid responses)	Unadjusted *p*-value	Bonferroni adjusted *p*-value (k = 11)
1: After the consultation, to what extent do you agree or disagree with the statement below: I learned something new about how to use my medication.	1: 4 (17%) 2: 1 (4%) 3: 4 (17%) 4: 4 (17%) 5: 11 (46%) No valid response: 0	**0.049**	0.539
2: After the consultation, to what extent do you agree or disagree with the statement below: I learned something new about why I should use my medication.	1: 6 (25%) 2: 6 (25%) 3: 5 (21%) 4: 2 (8%) 5: 5 (21%) No valid response: 0	0.506	-
3: After the consultation, to what extent do you agree or disagree with the statement below: I got answers to what I was wondering about.	1: 2 (8%) 2: 1 (4%) 3: 3 (13%) 4: 1 (4%) 5: 17 (71%) No valid response: 0	**< 0.001**	**< 0.011**
4: After the consultation, to what extent do you agree or disagree with the statement below: I received help to prevent or alleviate side effects.	1: 5 (22%) 2: 3 (13%) 3: 6 (26%) 4: 3 (13%) 5: 6 (26%) No valid response: 1	0.768	-
5: After the consultation, to what extent do you agree or disagree with the statement below: I developed new concerns during the consultation	1: 19 (83%) 2: 3 (13%) 3: 0 (0%) 4: 1 (4%) 5: 0 (26%) No valid response: 1	**< 0.001**	**< 0.011**
6: After the consultation, to what extent do you agree or disagree with the statement below: The consultation made me less worried.	1: 3 (13%) 2: 1 (4%) 3: 5 (21%) 4: 7 (29%) 5: 8 (33%) No valid response: 0	**0.044**	0.484
7: After the consultation, to what extent do you agree or disagree with the statement below: I received good advice on how to remember to take my medication.	1: 5 (21%) 2: 1 (4%) 3: 8 (33%) 4: 2 (8%) 5: 8 (33%) No valid response: 0	0.377	-
8: To what extent do you agree or disagree with the following statement: The consultation was useful.	1: 2 (8%) 2: 0 (0%) 3: 5 (21%) 4: 2 (8%) 5: 15 (63%) No valid response: 0	**< 0.001**	**< 0.011**
9: To what extent do you agree or disagree with the following statement: The pharmacist communicated professional knowledge in a clear and understandable way.	1: 0 (0%) 2: 0 (0%) 3: 0 (0%) 4: 2 (9%) 5: 21 (91%) No valid response: 1	**< 0.001**	**< 0.011**
10: To what extent do you agree or disagree with the following statement: The consultation helped address the challenges that were discussed.	1: 3 (13%) 2: 1 (4%) 3: 2 (9%) 4: 7 (30%) 5: 10 (43%) No valid response: 1	**0.018**	0.198
11: Overall, how satisfied or dissatisfied are you with this migraine consultation? (1 = very unsatisfied, 6 = very satisfied)	1: 0 (0%) 2: 0 (0%) 3: 2 (8%) 4: 3 (13%) 5: 2 (8%) 6*: 17 (71%) No valid response: 0	**< 0.001**	**< 0.011**

One sample Wilcoxon signed-rank tests against the scale midpoint; unadjusted and multiplicity adjusted p-values are shown, significant values in bold text

* 6-point Likert scale for the Overall question

#### Implementation of pharmacist recommendations and changes in migraine status

**Fig. 2:**
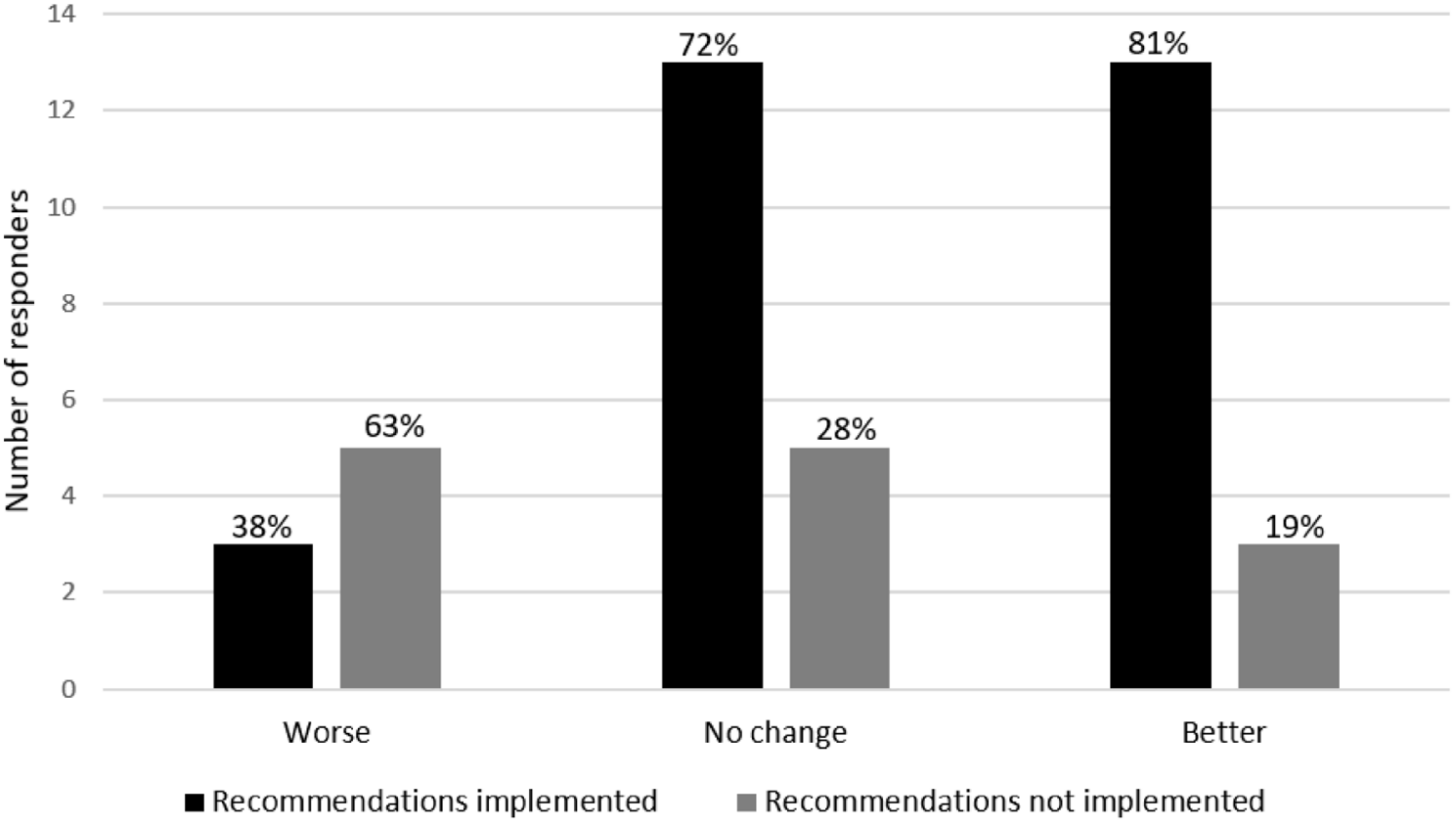
Number of respondents according to self-reported implementation of recommendations from the first consultation and self-reported migraine status at follow-up

During the follow-up consultations, patients were asked whether they had implemented any recommendations from the first consultations, whether their migraine status had changed, and if they had experienced a change in absence from work, school or other daily activities. Non-responders from the question on recommendations implemented were coded as “no”. Free text responses on question about migraine status since last consultation where coded as either “getting worse”, “no change”, or “getting better”, and absence was coded as either less absence, more absence or no change. 69% reported to have implemented at least one recommendation from the pharmacist at the first consultations, 38% reported to have an improved migraine status, but only 11% reported to have less absence from work, school or other daily activities. Participants who implemented recommendations reported fewer and/or less severe migraine attacks; however, this difference did not reach statistical significance with a chi square test (*p* = 0.085). These results are shown in Fig. 2. These findings reflect early indicators of service uptake and behavioural response following pharmacist counselling.

The recommendations most frequently implemented by patients concerned the administration of the medication (50%), more realistic expectations for treatment effect (41%), and dosing of medication (35%).

### Preliminary assessment of hypotheses

Although this was not a mainly hypothesis-driven efficacy trial, preliminary trends were compared with the study’s research expectations:

- Pharmacists perceived migraine consultations as beneficial and professionally meaningful, despite time constraints and organizational challenges. Pharmacist training prior to the consultations enhanced their confidence in migraine counselling. (Consistent with study expectations and H1).
- Gaps in patient medication knowledge were identified, particularly regarding interactions, side effects and treatment expectations. There was no significant difference in medication related knowledge gaps between new and experienced users. (Consistent with study expectations and H2).
- Patients reported high motivation for medication use and had generally positive consultation experiences, although these findings should be interpreted cautiously given the low response rate (Consistent with study expectations and H3).
- Patients who implemented recommendations from the first consultation showed trends toward self-reported changes in migraines status. Absence from work, school or social activities decreased in 11% of participants from the first consultations to the follow-up consultation. (Consistent with study expectations and H4).

### Summary of key findings

The findings highlight both implementation-related outcomes and service outcomes associated with pharmacist-led migraine consultations in community pharmacies. From an implementation perspective, pharmacists reported that the consultations were professionally meaningful and allowed them to utilize their clinical knowledge more fully. At the same time, they identified practical conditions that may influence service delivery, including the need for sufficient time for pharmacist training, adequate time for consultations, and appropriate financial support. Pharmacists also described patient information needs, particularly regarding correct triptan use, medication timing, and migraine self-management, as well as the opportunity to provide broader non-pharmacological advice.

Service outcomes were reflected in the patient-reported data. The responding patients were motivated to participate in the consultations and reported generally positive experiences. A majority of patients implemented at least one recommendation following the first consultation, and some reported perceived improvements in migraine status and reduced absence from daily activities. Although participants who implemented recommendations reported fewer or less severe migraine attacks, this difference did not reach statistical significance.

The small number of observations from follow-up consultations, absence of a control group, and reliance on self-reported measures mean that findings must be interpreted cautiously. The results primarily serve to guide further development, refine the intervention, and identify requirements for a future larger-scale or controlled trial.

## Discussion

A high proportion of the patients reported more than eight attacks in the past 30 days, and more than half of the participants experienced absence from work, school or social activities, indicating a high frequency of migraine episodes and significant disease burden. This highlights the profound functional and socioeconomic impact of migraine, particularly among new users who may have less established treatment routines, and underscores the importance of low-threshold, pharmacist-led migraine consultations as an accessible support service.

This pilot study suggests that a pharmacist-led migraine consultation service in community pharmacies may represent a promising approach within community pharmacy practice, highlighting implementation-related opportunities and challenges when introducing structured counselling services. The structured consultations helped address well-documented gaps in medication knowledge, supported safer and more effective self-management, and reinforced pharmacists’ role as accessible healthcare providers within primary care. While pharmacist-led interventions are well-established in other chronic conditions [11], this study adds novel insights into their relevance in migraine, a neurological disorder where self-management, appropriate medication use, and timely intervention are essential to prevent worsening symptoms and complications, including medication-overuse headache (MOH) [20].

### Interpretation of key findings

Across consultations, pharmacists identified medication related gaps among migraine patients, including uncertainty about when and how to take triptans, appropriate dosing intervals, differentiation between acute and preventive treatment, and realistic expectations for treatment outcomes. This was consistent among both experienced and newly diagnosed users. Many patients expressed fear of side effects or addiction to triptans and were unsure about switching between different triptan formulations. This aligns with previous research showing that incorrect timing, underdosing or overdosing, and misunderstanding of pharmacological action contribute to ineffective therapy [21].

Importantly, 69% of patients who attended the follow up consultation reported implementing at least one recommendation from their first consultation. A majority of these patients experienced an improvement in migraine status, although this did not reach statistical significance. Additionally, patients reported positive experiences with many aspects of the consultations. This behavioral change indicates that pharmacist counselling may empower patients to manage their migraine more effectively and suggest that such services may influence patient self-management and engagement with care.

Pharmacists described the consultations experience as professionally valuable. Many expressed that this service allowed them to utilize their pharmacological expertise more fully and strengthened their clinical identity beyond dispensing. Patients reportedly appreciated being listened to, receiving personalized advice, and having their concerns taken seriously, enhancing trust in pharmacists as health advisors. Together, these findings point not only to patient-level effects but also to factors related to the implementation of the service within routine pharmacy practice, including professional role development and perceived value of the service.

Although this study was not prospectively designed within a specific implementation framework, the findings can be interpreted through the lens of CFIR. Determinants were identified across multiple domains, including patient needs and knowledge gaps in the outer setting, organisational constraints such as time and workflow within pharmacies (inner setting), pharmacists’ professional confidence and role perceptions (characteristics of individuals), and practical aspects of service delivery such as consultation structure and follow-up procedures (intervention characteristics/process). Together, these multilevel factors appear to have shaped the uptake and execution of the consultations, as well as patients’ engagement with follow-up. While the present study does not constitute a formal implementation evaluation, applying CFIR as an interpretive framework helps contextualize these findings within a broader implementation process and may inform further development of pharmacist-led migraine services.

### Comparison with existing literature

The findings of this study support and extend existing literature on pharmacist-led interventions in chronic conditions such as asthma, cardiovascular disease, and diabetes, where pharmacists may contribute to improved medication use, treatment outcomes, and patient satisfaction [22]. However, migraine requires a different therapeutic approach, as treatment efficacy depends on fast recognition of attack onset, appropriate timing of medication, and informed switching between acute and preventive strategies. Unlike chronic medication regimens, migraine treatment is dynamic and heavily reliant on patient decision-making. This highlights the importance of educational interventions delivered at accessible settings such as community pharmacies.

Medication-overuse headache (MOH) remains one of the most significant complications of migraine management [23]. Studies have shown that excessive or unsupervised use of acute medications, especially triptans and NSAIDs, contributes to MOH and worsening headache patterns [24]. Our findings reflect this: pharmacists identified knowledge gaps and observed different misunderstandings regarding medication use among patients. By providing structured migraine consultations, pharmacists may help address risk factors toward MOH through early education and reinforcement of medication guidelines, an effect that warrants investigation in future controlled trials.

While pharmacist-led services are widely evaluated in metabolic diseases, evidence in neurological disorders remains scarce. Studies in depression, epilepsy, and chronic pain suggest that pharmacists can play a supportive role in monitoring treatment, educating patients, and collaborating with multidisciplinary teams when supported by appropriate training [25–27]. This pilot adds new evidence to this field, demonstrating that pharmacist-led consultations for migraine is not only well received by patients and pharmacists but also logistically possible within Norwegian community pharmacy settings.

### Implications for pharmacy practice and primary healthcare

This study reinforces the evolving role of the pharmacist from a product-oriented dispenser to a patient-centred healthcare provider. Counselling services such as those tested in this study could bridge gaps in migraine care by providing immediate support, guidance on medication management, and early identification of patients requiring medical review or preventive therapy. This approach aligns with current health policy goals emphasising task-sharing, decentralised care, and strengthened primary healthcare systems [28].

For potential wider implementation, structured models may be required, including appointment-based consultations, clearer referral pathways with general practitioners, defined remuneration, and digital documentation tools.

### Methodological considerations and limitations

As a pilot study, the primary aim was to explore implementation-related experiences and early service outcomes rather than effectiveness; therefore, no formal sample size or power calculation was performed prior to study initiation. A key limitation is the small number of patients who attended both consultations, which limits statistical power and generalizability. Attrition between consultations may have been influenced by migraine severity, time constraints, or the absence of systematic follow-up reminders. Furthermore, the interval between the first and second consultation, typically two to eight weeks, may have been too short to capture sustained changes in migraine outcomes, particularly for patients initiating preventive treatment. Improvements reported at follow-up may also have been influenced by medication changes occurring around the time of the first consultation, making it difficult to distinguish the effects of counselling from pharmacological treatment effects.

The few responses in the patients’ motivation and consultation experience questionnaire reflect a low voluntary response rate rather than a predefined sampling procedure. This introduces potential response bias, as patients with particularly positive or negative experiences may have been more likely to respond. These findings should therefore be interpreted cautiously. In addition, reliance on self-reported questionnaires introduces the potential for recall and social desirability biases [29].

Another limitation is that follow-up information was collected within the consultation context and, in some cases, by the same pharmacist who delivered the counselling. This may have introduced reporting or social desirability bias, potentially influencing patient responses and reported implementation of recommendations. However, structured questionnaire items were used to reduce subjective interpretation during data recording.

A further limitation relates to variability in pharmacists’ prior knowledge and experience, which may have affected the consistency and quality of counselling despite completion of a standardised training programme. The absence of a control group prevents causal conclusions.

All authors are female and trained pharmacists. Reflexivity was therefore explicitly considered, particularly in the qualitative component of the study, given the potential influence of professional background on study design, data collection, and interpretation of findings—especially assessments of experiences relevant to early service implementation. Given the exploratory and pilot nature of the study, qualitative data collection was not guided by a predefined aim of achieving data saturation, but rather by the purpose of capturing a range of experiences relevant to implementation of the service. Potential bias was mitigated through the use of structured data collection tools, predefined analytical procedures, and triangulation of quantitative and qualitative data. The three senior researchers involved in the study have 15–25 years of experience in quantitative and qualitative research methods.

### Future research directions

Future studies should use larger, multi-centre designs with control groups, validated instruments to collect patient-reported outcomes and more extended observation periods to assess sustained effects on adherence, attack frequency, initiation of preventive therapy, and risk reduction for MOH. Health-economic analysis should determine whether pharmacist-led consultations reduce costs related to emergency visits, GP appointments, and sick leave.

Digital solutions, including secure appointment booking, electronic headache diaries, and telepharmacy consultations, can be tested as tools to enhance continuity and documentation. Comparative studies between pharmacist-, physician-, and nurse-led counselling models could clarify optimal roles within multidisciplinary migraine care. Finally, future implementation efforts may require national-level strategies, including reimbursement systems, accreditation programmes, and integration into routine pharmacy workflows.

## Conclusion

This pilot study suggests that pharmacist-led migraine consultations may support patient-centred migraine care within community pharmacy practice, particularly when adequate financing allows pharmacies to allocate sufficient time. Responding patients reported generally positive consultation experiences, a majority reported implementing at least one recommendation, and many reported perceived improvements in migraine status after the consultations. Pharmacists described that the counselling service was professionally meaningful and strengthened their perceived role and clinical identity within primary healthcare, and that the structured model helped address important information gaps.

Given the limited number of follow-up consultations and patient reported consultation experience questionnaires, and the reliance on self-reported outcomes without a control group, these findings should be interpreted as preliminary signals rather than evidence of effectiveness. Nevertheless, the consistently positive perceptions among both patients and pharmacists indicate potential value for an accessible, patient-centred service within primary care.

Future research should focus on larger, multi-site studies with a priori planned sample sizes, use of validated instruments, extended follow-up periods, inclusion of control groups, and objective outcome measures to confirm and further explore these preliminary findings.

Community pharmacists may represent an underutilised resource in migraine care by delivering structured consultations that support appropriate medication use and patient engagement, and this pilot study highlights the benefits and implementability of such services as a complement to existing headache care pathways. These findings should be interpreted as exploratory and hypothesis-generating, providing a foundation for future controlled and implementation-focused studies.

## Data Availability

The authors recognize the journal’s commitment to data transparency. Owing to organizational data governance policies at Apotek 1, the structured consultation questionnaire and interview guide cannot be publicly shared but are available upon reasonable request from Apotek 1 and can be provided to editors and reviewers during the review process.
